# Upper Motor Neuron Signs in the Cervical Region of Patients With Flail Arm Syndrome

**DOI:** 10.3389/fneur.2021.610786

**Published:** 2021-02-15

**Authors:** Yingsheng Xu, Junyi Chen, Shuo Zhang, Dongsheng Fan

**Affiliations:** ^1^Department of Neurology, Peking University Third Hospital, Beijing, China; ^2^Beijing Key Laboratory of Biomarker and Translational Research in Neurodegenerative Diseases, Peking University Third Hospital, Beijing, China

**Keywords:** flail arm syndrome, upper motor neuron, triple stimulation technique, pectoralis tendon reflex, amyotrophic lateral sclerosis

## Abstract

**Objective:** We investigated upper motor neuron (UMN) signs in the cervical region in a Chinese clinic-based cohort of patients with flail arm syndrome (FAS) by clinical examination and neurophysiological tests such as triple stimulation technique (TST) and pectoralis tendon reflex testing.

**Methods:** A total of 130 consecutive FAS patients from Peking University Third Hospital underwent physical examination and neurophysiological tests at baseline and 3 months, 6 months, 9 months, and 12 months later. Pyramidal signs, pectoralis tendon reflex and TST results were evaluated to estimate the function of cervical spinal UMNs.

**Results:** At the first visit, weakness of the bilateral proximal upper limbs was found in 99 patients, while weakness of a single proximal upper limb was found in 31 patients. There were 49 patients with tendon hyperreflexia, 42 patients with tendon hyporeflexia and 39 patients with tendon areflexia. All except 4 of the patients had brisk pectoralis tendon reflex. The UMN score of the cervical region was 1.7 ± 0.4, and the lower motor neuron score of that region was 3.5 ± 0.3. The TST_test_/TST_control_ amplitude ratio was 65.7 ± 7.5%. The latency of quantitative detection of the pectoralis tendon reflex was 7.7 ± 1.2 ms. In the follow-up study, the UMN score and the TST_test_/TST_control_ amplitude ratio decreased, while the lower motor neuron score increased, and the latency of quantitative detection of the pectoralis tendon reflex remained steady.

**Conclusion:** Although the signs of cervical spinal UMN dysfunction in patients with FAS were often concealed by muscle atrophy in the progression of the disease, TST and pectoralis tendon reflex could reveal it.

## Introduction

Flail arm syndrome (FAS), a variant of amyotrophic lateral sclerosis (ALS), is characterized by progressive proximal weakness and muscle wasting of the upper limb (1–5). The symptoms may be confined to the cervical region, without functional involvement of the lower limb, thoracic muscles or bulbar muscles, for a period of at least 12 months. Meanwhile, signs of upper motor neuron (UMN) lesions of the cervical region in FAS patients are rare. Therefore, it is difficult to differentiate FAS from other lower motor neuron syndromes in clinical manifestations. Meanwhile, the triple stimulation technique (TST) (6–9), a quantitative and reliable method for assessing UMN loss, and the pectoralis tendon reflex, whose reflex center is broad (C5-T1), could be used to reveal covert UMN lesions (10, 11). We performed TST and pectoralis tendon reflex tests in FAS patients to confirm the evidence of cervical spinal UMN impairment.

## Materials and Methods

### Subjects

This clinic-based cohort study was approved by the institutional ethics committee of Peking University Third Hospital (No.2014110) and written informed consent was obtained from each patient before they were recruited for the study.

From January 2010 to December 2019, all patients who visited the clinic for ALS at Peking University Third Hospital and were diagnosed with sporadic ALS were recruited. The inclusion criteria included weakness of the upper limbs as an initial symptom. The time of disease onset was defined as the date when patients noticed their symptoms for the first time. The diagnosis of FAS was made when the patient presented muscle weakness in the upper limbs, without functional involvement of bulbar muscles or lower extremities for at least 12 months, according to the diagnostic criteria (5, 12, 13), on the patients' first visits to our clinic and during the follow-up. Patients with hypertonia were excluded. If the course of the disease was <12 months on the patients' first visits, the diagnosis of FAS might be made during the follow-up until they met the diagnostic criteria. Those who developed functional involvement of bulbar muscles or lower extremities within 12 months after onset during follow-up were excluded. These patients had no epilepsy, refractory hypertension, cardiac pacemakers or other implanted electronic devices.

### Methods

#### Baseline Data

We collected baseline data, including demographics, age at symptom onset, and age at diagnosis, which were confirmed by the Declaration of Helsinki.

We recorded initial symptoms, positive signs of physical examination and results of neurophysiological examination (TST and quantitative pectoralis tendon reflex tests).

#### Follow-Up Study

Each patient in the study was given a follow-up evaluation every 3 months to collect the physical signs and the results of the TST and quantitative pectoralis tendon reflex tests. Signs of impairments of UMNs and lower motor neurons (LMNs) were recorded, especially in the cervical region.

#### Clinical Evaluation

All patients were interviewed and examined carefully. Reflexes of the cervical region included the pectoralis tendon reflex, biceps reflex, elbow reflex and radial reflex. LMN signs included muscle weakness, atrophy, fasciculation, hypomyotonia, hyporeflexia and areflexia. UMN signs were identified by the presence of muscle weakness, hyperreflexia with pathological reflex spread, spasticity, clonus, preserved reflexes in weakly wasted limbs, Babinski's signs and so on. Muscle weakness due to UMN impairment is obscure and usually presents as clumsy, worse coordination and slower compound movement. The scores of UMN and LMN lesions were recorded according to the assessment standard (14–16) by the following scale: 0 = no involvement; 1 = definite but trace involvement; 2 = moderate involvement; and 3 = significant and severe involvement. The UMN or LMN score of the cervical region was calculated by summating the scores in each upper limb, ranging from 0 to 6. The scoring system was used to quantify clinical UMN and LMN involvement.

#### Neurophysiological Examination

The tests were performed with a Keypoint electromyography (EMG) machine (Medtronic, USA), a MagPro magnetic stimulator, a circular coil, C-100 (Medtronic, USA), with an outer diameter of 105 mm and an inner diameter of 20 mm and a reflex hammer that included a sensor system controlled by a spring switch.

Motor evoked potential (MEP) and TST test (6–9, 17–19): Transcranial magnetic stimulation (TMS) was performed before the TST test according to international guidelines (20), using a magnetic stimulator and a circular coil with recording from the abductor digiti minimi (ADM) on each side. Nerve conduction studies were performed using standard techniques. Bandpass filtering was between 2 Hz and 10 kHz. Central motor conduction time (CMCT) was recorded and analyzed subsequently. During the TST tests, the subject was lying on a bed in the supine position. Measurement of the evoked response over the ADM is performed following stimulation of the motor cortex, wrist and Erb's point in succession. It is performed by first delivering a magnetic stimulus to the motor cortex followed by supramaximal electrical stimulation of the ulnar nerve supplying the ADM at the wrist such that the descending corticomotoneuronal volley is ‘collided' out by the antidromic ulnar action potentials. Collision takes place along the proximal segment of the ulnar nerve at the upper arm. A third stimulus is subsequently delivered to Erb's point (axilla) after an appropriate delay, eliciting a highly synchronized motor response in those fibers in which the collision had occurred. Thus, three stimuli were given, leading to two collisions (cortex-wrist, wrist-Erb's point). The amplitude and area of this test compound muscle action potential (CMAP) response are compared with the response induced by the conditioned TST paradigm (Erb's point-wrist-Erb's point stimulation). The derived TST amplitude ratio allows the estimation of a proportion of surviving corticospinal motor neurons. The more severely affected side was included in the analysis.

Quantification of the pectoralis tendon reflex test (10, 11): The subject was lying on bed in the supine position. The forearm was placed in the position of abduction, with an angle of 30° between the upper arm and the trunk. The recording electrode was placed in the belly of the pectoralis major (~5–7 cm below the midclavicular line), and the reference electrode was placed in the sternum. The examiner placed his left thumb on the patient's pectoralis major tendon in the deltopectoral groove and held the hammer in the right hand. Knocking the tendon, the examiner could see abduction of the shoulder and feel the movement of the tendon. The reflex could also be recorded in the EMG machine simultaneously. The reflex is elicited 3–5 times successively with at least 5 s delay each, and the graph is recorded with clear latency and amplitude.

### Statistical Analysis

Data analyses were performed using the SPSS V.21.0 software package, and significance was set at a level of 5%. Normality tests were performed for all continuous variables. Parametric tests (one-way analysis of variance (ANOVA) or Student's *t*-test) or non-parametric tests (the chi-square test, the Kruskal-Wallis one-way ANOVA by ranks, or the Mann-Whitney *U* test) were used to compare differences between groups as appropriate. The matched *t-*test and ANOVA for repeated measures were performed to test the significance of differences for variables during follow-up. Normally distributed data are expressed as the means ± standard errors, and the UMN and LMN scores are expressed as the means ± standard errors and range.

## Results

### Baseline Characteristics

From January 2010 to December 2019, 1,500 patients with sporadic ALS were recruited in our ALS cohort. Among them, 146 suspected FAS patients were initially recruited for this study. In these patients, 130 eventually met the diagnostic criteria for FAS, and during the follow-up, 16 were excluded because of functional involvement of bulbar muscles or lower extremities earlier than 12 months after onset. The ratio of males to females was 5:1 (109:21). The age range was 28 to 77 years, the mean age was 52.2 ± 6.1 years, and the disease duration was 3–92 months.

Of the 130 FAS patients, 99 patients had bilateral upper limb weakness, 18 patients had right upper limb weakness, and 13 patients had left upper limb weakness at the first visit. The patients with unilateral weakness were at the early stages of the disease (duration <12 months). According to the revised El Escorial criteria (12, 21), 3 patients were classified as definite, 12 patients were probable ALS, 25 were probable supported by laboratory findings, 68 were possible, 8 were suspected (LMN signs only) and 14 could not be classified.

The patients were divided into four groups according to the disease course: Group 1, disease duration <6 months (25 cases); Group 2, disease duration 6–12 months (40 cases); Group 3, disease duration 12–24 months (36 cases); and Group 4, disease duration >24 months (29 cases). The patients' demographic characteristics were shown in Table 1.

**Table 1:** Demographic characteristics of the FAS patients.

**Groups**	**Number of cases (*n*)**	**Age (years)**		**Sex (M/F)**
		**Mean ± SD**	**Range**	
Group 1	25	49.7 ± 5.5	45–68	20/5
Group 2	40	52.3 ± 6.2	49–72	34/6
Group 3	36	51.6 ± 7.2	28–77	29/7
Group 4	29	54.3 ± 9.1	48–66	26/3

### Clinical Manifestation

There were 32 patients with hyperactive sucking reflex or jaw jerk, 49 patients with tendon hyperreflexia in the upper limbs (biceps reflex, elbow reflex or radial reflex), 42 patients with hyporeflexia and 39 patients with areflexia. Brisk pectoralis tendon reflex and pathological reflex spread were observed in 126 patients. Absent abdominal reflexes were found in 72 cases. There were 122 patients with tendon hyperreflexia in the lower extremities and 75 patients with extensor plantar responses. Clumsy and poor coordination of bilateral proximal upper limbs were seen in 33 patients, and poor coordination of unilateral proximal upper limb was seen in 17 cases. The UMN score of the cervical region was 1.7 ± 0.4 (0–4), while the LMN score of the cervical region was 3.5 ± 0.3 (0–6). There were significant differences in the UMN and LMN scores among the four groups (*P* < 0.05).

### TST and Quantification of Pectoralis Tendon Reflex Test

**Figure 1:**
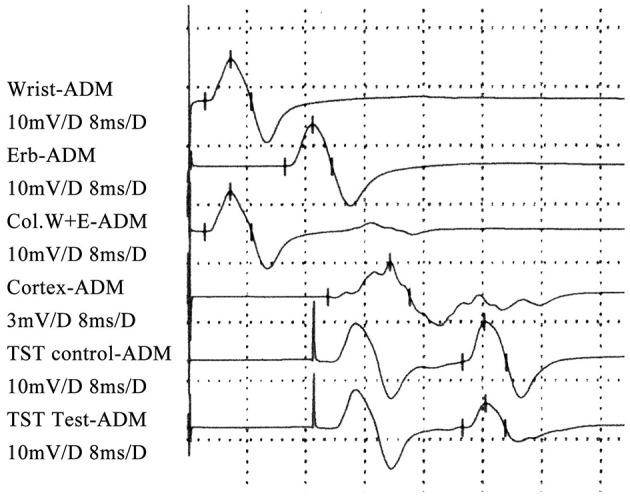
TST test of an FAS patient: the TST_test_/TST_control_ amplitude ratio decreased.

**Figure 2:**
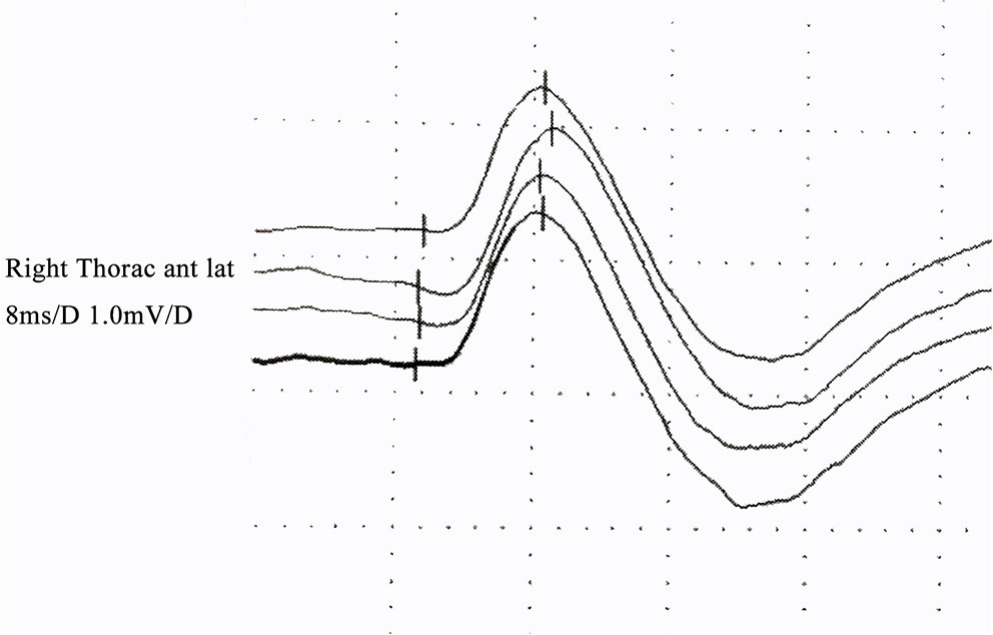
Quantitative pectoralis tendon reflex in an FAS patient: the latency and amplitude were normal.

The TST_test_/TST_control_ amplitude ratio was 65.7 ± 7.5% (Figure 1). The latency and amplitude of quantitative detection of the pectoralis tendon reflex were 7.7 ± 1.2 ms and 1.2 ± 0.1 mV, respectively (Figure 2). The TST amplitude ratio of patients with longer disease duration was significantly lower than that of subjects in the early stages (*P* < 0.05). However, there was no significant difference in the latency of the pectoralis tendon reflex among the four groups (*P* = 0.65). Reference values of CMCT, TST amplitude ratio and quantitative detection of the pectoralis tendon reflex were obtained from previous studies performed in our EMG laboratory (18, 22, 23). The following values were established as normal: CMCT < 7.6 ± 1.2 ms; TST amplitude ratio > 93.0 ± 2.7 %; the latency and negative peak amplitude of pectoralis tendon reflex were 8.2 ± 1.0 ms and 1.0 ± 0.2 mV respectively. The TST amplitude ratio was abnormal in 102 patients, while the CMCT was prolonged in only 65 patients. 67.9% (55/81) FAS patients without UMN signs in the upper limbs had an abnormal TST amplitude ratio, and 97.5% (79/81) of them had brisk pectoralis tendon reflex or pathological reflex spread.

### Follow-Up Study

Three months later, there were 43 patients with hyperactive sucking reflex or jaw jerk, 42 patients with tendon hyperreflexia in the upper limbs, 47 patients with tendon hyporeflexia and 41 patients with tendon areflexia. Brisk pectoris tendon reflexes and pathological reflex spread were observed in 127 patients. Absent abdominal reflexes were found in 86 cases. There were 128 patients with tendon hyperreflexia in the lower extremities and 84 patients with extensor plantar responses. The UMN score of the cervical region was 1.4 ± 0.2 (0–4). The LMN score of the cervical region was 4.3 ± 0.5 (1–6). In the TST, the TST_test_/TST_control_ amplitude ratio was 58.7 ± 6.1%. The latency and amplitude of quantitative detection of the pectoralis tendon reflex were 7.9 ± 1.1 ms and 1.2 ± 0.2 mV, respectively.

**Figure 3:**
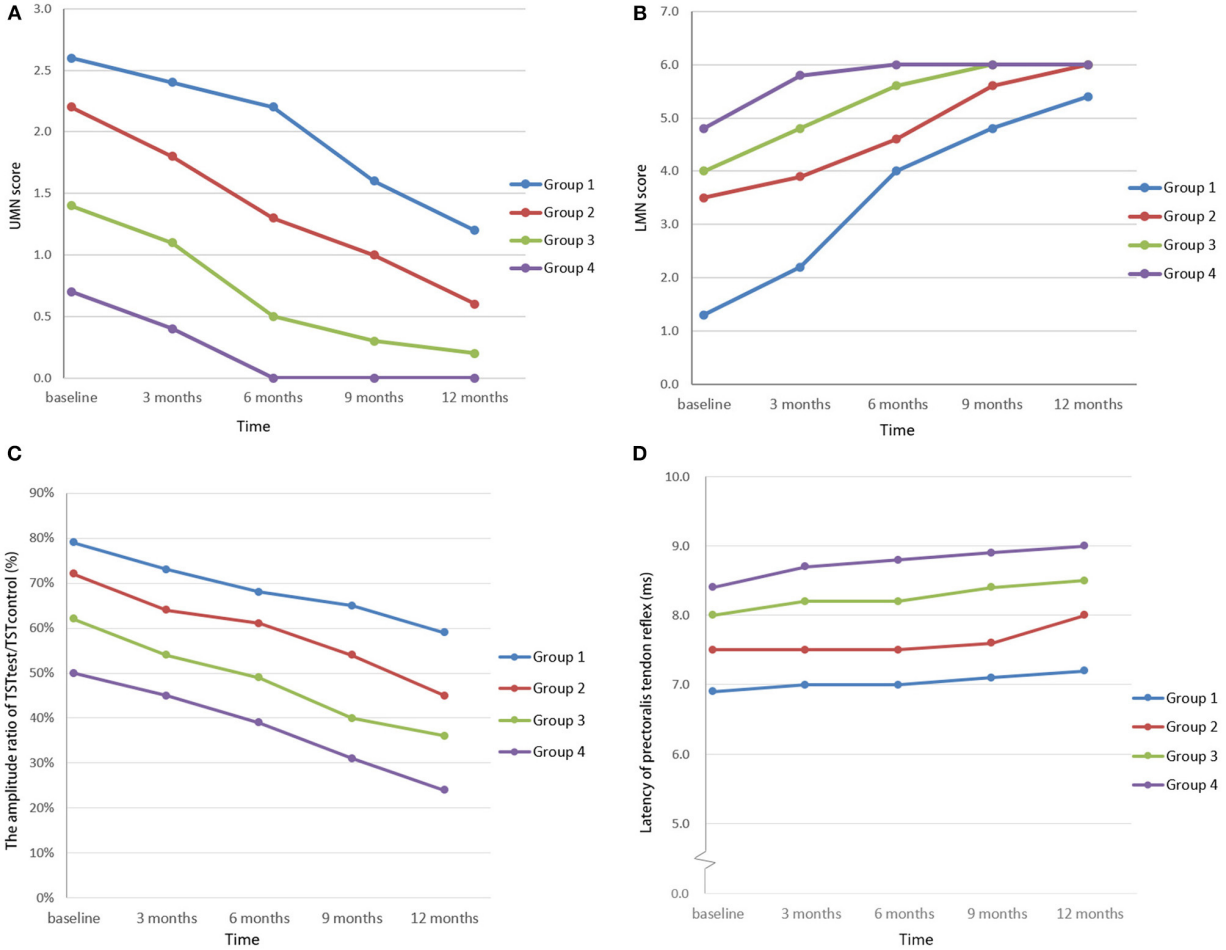
UMN scores of the cervical region **(A)**, LMN scores of the cervical region **(B)**, TST_test_/TST_control_ amplitude ratios **(C)**, and latencies of quantitative detection of the pectoralis tendon reflex **(D)** among FAS patients in the study. In the follow-up study, the UMN score and the TST_test_/TST_control_ amplitude ratio decreased, in contrast to the increased LMN score, while the latency of quantitative detection of the pectoralis tendon reflex remained almost unchanged. (Patients were divided into four groups according to the length of the disease course: group 1: disease duration < 6 months; group 2: disease duration 6–12 months; group 3: disease duration 12–24 months; and group 4: disease duration >24 months).

During the 1-year follow-up (five cases lost), the tendon reflexes and the UMN score declined gradually, the TST_test_/TST_control_ amplitude ratio decreased, while the LMN score increased and the pectoralis tendon reflex remained almost unchanged (Figures 3A–D). There were significant decreases in UMN scores and TST amplitude ratios in the four groups at every time point (*P* < 0.05) and significant increases in LMN scores at baseline, 3 months and 6 months. There was no significant difference in the latency of the pectoralis tendon reflex among the four groups (*P* = 0.72). There were only two patients with brisk tendon reflex in the upper limbs, while the TST amplitude ratio was abnormal in 120 patients. The pectoralis tendon reflexes remained unchanged. Five of eight patients with pure LMN syndrome had an abnormal TST or pectoralis reflex.

## Discussion

FAS presents with progressive weakness and muscle wasting in the proximal upper limbs at onset; it may gradually spread to other regions. Signs of cervical UMN impairment are invisible and may be concealed by LMN symptoms such as muscle atrophy, therefore, the characteristics of UMN signs in FAS patients are not described well. There are some studies of it, and the results were different without consensus. Wijesekera et al. found that 41.8% of FAS patients had at least one UMN sign in the flail region (4). It was reported that approximately 70% (14/20) of FAS patients developed UMN signs in the lower limbs (3), and 50% (9/18) had UMN signs in the cervical region (5). Yang et al. found no obvious UMN signs and no abnormal CMCT in FAS patients. However, the sample sizes were limited, and only six FAS patients were included (24). The striking result of our study is that careful physical examination and neurophysiological tests can reveal UMN deficits in the early stage and the follow-up study.

In this study, we found that impaired coordination due to muscle weakness was present in the early stage of FAS (disease duration <6 months) without EMG evidence of LMN loss, which was a sign of UMN lesions and was hardly recognized by the patients themselves (25). As the disease progresses, weakness and wasting of muscles due to LMN impairment gradually became obvious, and the patients tended to be diagnosed with LMN syndromes. LMN lesions lead to imperfection of the spinal motor system in the cervical region that reduces the active signals of the UMN system, so it is difficult to elicit tendon hyperreflexia. It is necessary to judge whether the tendon reflex is relatively brisk on the basis of muscle weakness and wasting, but “relatively brisk” (26) is a rather subjective clinical judgment. Therefore, it is difficult to identify UMN lesions in the cervical region by usual clinical examination alone.

On the other hand, the pectoralis tendon reflex and TST amplitude ratio are hardly affected by LMN signs compared with standard neurophysical examination.

In our study, the TST_test_/TST_control_ amplitude ratio decreased as the disease duration increased, suggesting progressive impairment of the pyramidal tract. TST is a sensitive method to detect subclinical UMN abnormalities in ALS patients by eliminating the effects of repetitive spinal motor neuron discharge, desynchronization and unstable response to stimuli in classical transcranial magnetic stimulation, which provides a method of evaluating UMN deficits (6). Furtula J et al. found that seven of 13 ALS patients had abnormal TST results, 57.1% (4/7) of them had no clinical UMN signs at the cervical region, and 66.7% (4/6) ALS patients without UMN signs at the cervical region had abnormal TST results (6). Wang et al. recently found that 27.3% (6/22) ALS patients without UMN signs had an abnormal TST ratio, and TST appeared to be a more accurate measure for detecting clinical and subclinical UMN dysfunction in ALS than conventional TMS (27). In our study, fifty five of 81 (67.9%) FAS patients without UMN signs in the upper limbs had an abnormal TST amplitude ratio initially, indicating UMN involvements. Some of these patients might have subclinical UMN dysfunction, which were detected by TST. While in the other patients with high LMN scores of the cervical region, the signs of UMN impairment might be concealed by LMN symptoms but could be detected by TST. It is hard to distinguish these two kinds of patients at the beginning and define the cut-off value of LMN scores. Thus, we recommend to perform TST in suspected FAS patients to find more evidence of UMN abnormality. Further studies will be required to evaluate the advantage of TST to detect UMN dysfunction in patients without UMN signs in the neurological examination.

At the same time, the results of the quantitative pectoralis tendon reflex test suggested that the pectoralis tendon reflex persisted and was rarely influenced by the duration of disease. The structural basis of the pectoralis tendon reflex is near the chest, and its reflex center is broader (C5-T1); therefore, it is not easily affected by muscle wasting and is involved late in the disease course. It has been reported that the pectoralis tendon reflex could be evidence of impairment of cervical UMN (10, 11, 28). Therefore, the pectoralis tendon reflex could be an alternative method to detect cervical UMN lesions in FAS patients.

We did not find data about the TST or pectoralis tendon reflex of FAS patients in previous studies. In this study, the percentage of FAS patients with UMN signs in the cervical region was 38% (49/130) according to standard physical examination initially and 1.6% (2/125) after 12 months of follow-up. However, the percentage was increased to 97% (126/130) according to the TST and pectoralis tendon reflex test. We have shown in this study that FAS patients with different initial symptoms have different courses: (1) In previous studies, FAS was associated with a longer diagnostic delay than classical limb onset ALS (1, 3). Hübers et al. found that the initial symptom most frequently manifested on the dominant side of the upper limb in most FAS patients (2). In our study, the patients with unilateral weakness were at the early stages of the disease (duration <12 months). We inferred that patients might recognize the abnormality and come to see doctors earlier because of the asymmetric strength of the arms when the unilateral arm was involved at onset. At that time, UMN signs could be identified easily by clinical examination, and then the impairment of UMN of the other side and LMN could be involved gradually. (2) When both arms were involved at the same time, patients could not perceive the abnormality and did not visit the doctors in a timely manner. Muscle wasting as a result of LMN impairment concealed UMN signs; hence, UMN signs could not be detected by clinical examination, while TST and pectoralis tendon reflex played important roles in evaluating UMN lesions.

This study has several limitations. First, although this study was one of the largest studies in FAS, the number of patients was still limited. Those patients with minimal signs might be underrepresented. Next, this was a clinic-based cohort study, and the results may be different from those of a population-based study. Further studies with more cases of FAS will be required to confirm the observations from our cohort.

The results of our study confirmed that cervical spinal UMNs were involved in FAS, which supports the theory that ALS is derived from anterograde degeneration of the motor cortex with UMN involvement in the early stage (29, 30).

## Data Availability Statement

The original contributions presented in the study are included in the article/supplementary material, further inquiries can be directed to the corresponding author/s.

## Ethics Statement

The studies involving human participants were reviewed and approved by the institutional ethics committee of Peking University Third Hospital (No. 2014110). The patients/participants provided their written informed consent to participate in this study.

## Author Contributions

DF conceived this study and provide financial support. YX and JC performed the experiments, analyzed the data, and wrote the manuscript. SZ provided supplementary data. All authors contributed to the article and approved the submitted version.

## Conflict of Interest

The authors declare that the research was conducted in the absence of any commercial or financial relationships that could be construed as a potential conflict of interest.
